# Southeastern China Boreal Winter Precipitation Anomalies are Dependent on Intensity of El Niño

**DOI:** 10.1038/s41598-019-53496-5

**Published:** 2019-11-22

**Authors:** Zongjian Ke, Xingwen Jiang, Zunya Wang

**Affiliations:** 1 Laboratory for Climate Studies, National Climate Center, China Meteorological Administration, Beijing, China; 2 Institute of Plateau Meteorology, China Meteorological Administration, Chengdu, Sichuan China

**Keywords:** Climate sciences, Atmospheric dynamics

## Abstract

Previous studies reported that boreal winter precipitation in southeastern China (SEC) tends to increase during El Niño. In this study, however, we find that most weak El Niño events are accompanied by below-normal precipitation in SEC, although strong El Niño events are accompanied by above-normal precipitation in SEC for both eastern Pacific El Niño and central Pacific El Niño. Both the cold SST anomalies in the western North Pacific (WNP) and the warm SST anomalies in the central tropical Pacific are important for the formation of anomalous anticyclone over the WNP, which favors above-normal precipitation over SEC by transporting more water vapor to SEC. The cold SST anomalies in the WNP only excite a weak anomalous anticyclone locally when the weak warm SST anomalies in the central tropical Pacific are accompanied by weak enhanced convection anomalies. In such condition, El Niño does not affect precipitation in SEC apparently.

## Introduction

El Niño and Southern Oscillation (ENSO), the strongest interannual variability source, exerts significant impacts on global climate^1–4^. ENSO exerts different impacts on East Asian climate in different phases and statuses^5–10^. In boreal winter, the impacts of El Niño and La Niña on precipitation are asymmetric^11^; only El Niño favors above-normal precipitation over southeastern China (SEC) by exciting the western North Pacific anomalous anticyclone (WNPAC) in the lower troposphere, which transports more water vapor to SEC by the southwesterlies^6,7,12–14^. Thus, El Niño is a critical predictor for SEC precipitation in boreal winter.

In the recent years, the different features and impacts of two types of El Niño received extensive attention^15–20^. The two types of El Niño have different impacts on SEC precipitation in winter. The eastern Pacific (EP) El Niño causes more precipitation compared with the central Pacific (CP) El Niño because the former can excite stronger Walker circulation and WNPAC^16^. In addition, El Niño could cause above-normal precipitation over SEC in positive phase of the Pacific Decadal Oscillation (PDO) compared to that in negative phase of PDO, because the El Niño-like decadal SST anomalies during the positive phase of PDO enhance the magnitude of SST anomalies of El Niño, which induces a stronger WNPAC^21^. This study implied the impact of El Niño on SEC precipitation may be dependent on the magnitude of El Niño.

Some studies indicated that the climate impact of El Niño is sensitive to its magnitude^22–24^. Then, the question is whether the SEC precipitation is sensitive to magnitude of El Niño. In this study, thus, we investigate the relationship between boreal winter precipitation over the SEC and El Niño, with a focus on the impacts of different types of El Niño with different intensity on precipitation anomalies.

## Results

### Precipitation variability

**Figure 1:**
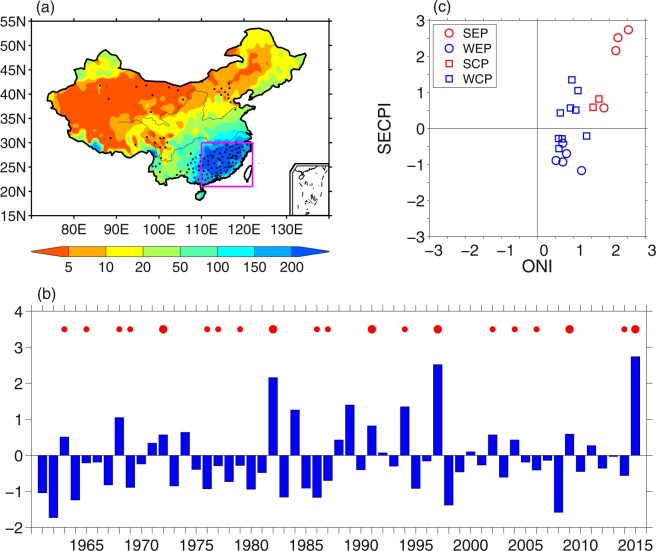
(**a**) Climatology of DJF accumulated precipitation (mm). (**b**) Normalized DJF southeastern China precipitation Index (SECPI). (**c**) Scatter plot of DJF SECPI and ONI for strong EP El Niño (SEP), weak EP El Niño (WEP), strong CP El Niño (SCP), weak CP El Niño (WCP) from 1961 to 2015. Black dots in (**a**) indicate stations with correlation coefficient between DJF precipitation and ONI from 1961 to 2015 exceeding 95% confidence level. Precipitation in the stations in the pink box in (**a**) are used to construct the SECPI in (**b,c**). The red dots in (**b**) indicate El Niño years and the size of the dots indicate the intensity of El Niño: bigger size of the dots indicating strong El Niño.

Boreal winter precipitation of China mainly concentrates in SEC, with a total above 150 mm (Fig. 1a). To quantify the precipitation in SEC, the SEC boreal winter precipitation index (SECPI) is derived by area-averaging the boreal winter precipitation amount over SEC, which is indicated by the box (110°E-122°E, 21°N-30°N) in Fig. 1a with 93 gauge stations being included. As shown in Fig. 1b, SECPI exhibits strong interannual variability, while a weak increase trend is also observed during the past 55 years. The winter precipitation over SEC is positively correlated with the oceanic Niño index (ONI) (Fig. 1a), with a correlation coefficient of 0.53. The precipitation over SEC tends to be above normal during El Niño years. This relationship has been well documented by previous studies^9,25^. However, it is worth noting that SECPI is below normal in about half of the El Niño years (Fig. 1c). The scatter plot of ONI and SECPI indicates that the SECPI is positively correlated with the intensity of ONI. The SECPI is positive for all the six strong El Niño events, and the years with the three largest SECPI are those with the strongest El Niño events. However, the SECPI is positive only for 5 out of the 14 weak El Niño events (35.7%), indicating that SEC precipitation tends to be below normal for weak El Niño.

Previous studies have indicated that the SEC boreal precipitation anomalies are different from different types of El Niño. Figure 1c shows that the occurrence rate of positive SECPI versus negative SECPI is 4/5 for the 9 EP El Niño events, while it is 7/4 for the 11 CP El Niño events. Of note is that the SECPI is positive for all the strong EP El Niño events, while it is negative for all the weak EP El Niño events. As for the 11 CP El Niño events, positive SECPI appears in two strong events, negative SECPI appears in 4 out of the 9 weak events. All these statistical analyses indicate that precipitation anomalies over SEC are more sensitive to the intensity of El Niño compared to its types.

### El Niño events’ impacts

**Figure 2:**
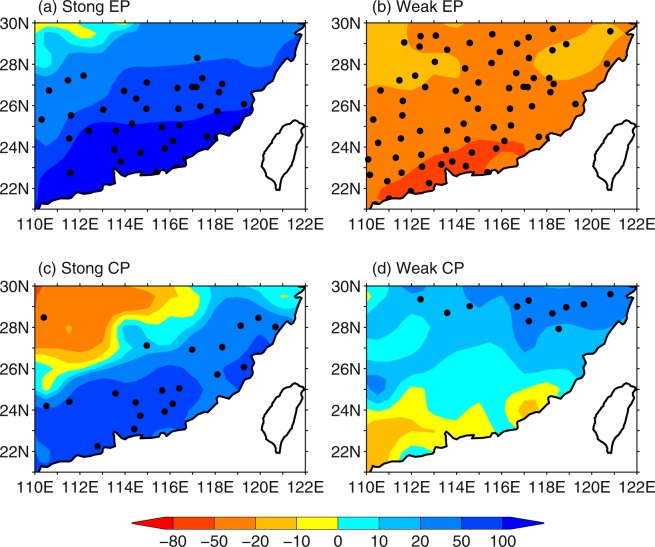
Composite percentage of DJF precipitation anomaly for (**a**) strong EP El Niño, (**b**) weak EP El Niño, (**c**) strong CP El Niño, and (**d**) weak CP El Niño. Stippling indicates the value of shadings exceeding 90% confidence level.

Figure 2 displays composite percentage of precipitation anomalies during El Niño events with different combinations of intensity and type. Most of SEC receives above-normal precipitation for strong EP and CP El Niño events, with magnitude of precipitation anomalies decreases from the southeast to the northwest. Precipitation is below normal in entire SEC for the weak EP El Niño events. Of note is that precipitation anomalies are totally opposite between strong and weak El Niño in the southeast coast areas, where larger precipitation anomalies are located. Again, the composite patterns show that the difference in intensity of El Niño plays a more important role in precipitation anomalies over SEC compared to its difference in types.

**Figure 3:**
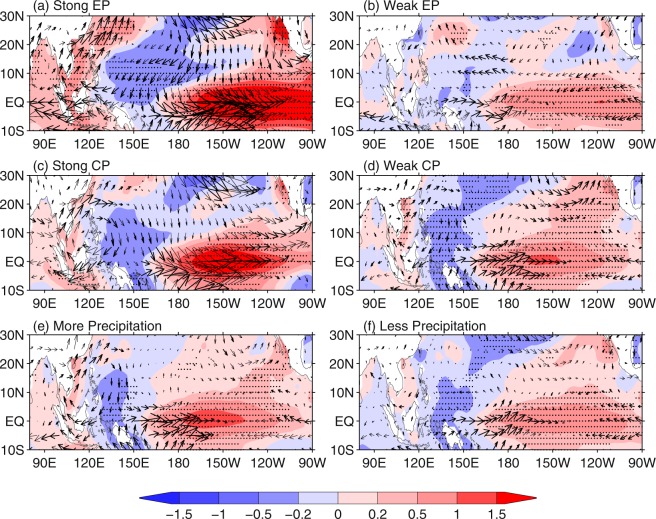
Composite anomalies of DJF 850-hPa wind (m s^−1^; vectors) and SST (°C; shadings) for (**a**) strong EP El Niño, (**b**) weak EP El Niño, (**c**) strong CP El Niño, and (**d**) weak CP El Niño. Composite anomalies of DJF 850-hPa wind (m s^−1^; vectors) and SST (°C; shadings) for (**e**) more and (**f**) less precipitation in SEC for weak El Niño events. Stippling indicates the value of shadings exceeding 90% confidence level. The bold arrows indicate values exceeding 90% confidence level in either the zonal or the meridional component.

Winter precipitation in SEC is closely linked to water vapor transported from the south, which is associated with an anomalous anticyclone in the lower troposphere over the western North Pacific (WNP)^6^. As shown in Fig. 3, a strong anomalous anticyclone is located over the WNP for the two types of strong El Niño and the southwesterlies prevail over SEC. In contrast, there is no apparent anomalous anticyclonic circulation over the WNP for weak EP El Niño, while a weak anomalous anticyclone is observed over the WNP for weak CP El Niño. The 850-hPa winds anomalies over SEC indicate that the strong anomalous anticyclone over the WNP during strong El Niño events favors more water vapor transported to SEC and above-normal precipitation in SEC. The anomalous wind features are basically consistent with the features of the precipitation anomalies. Of note is that the anomalous anticyclone is not accompanied by above-normal precipitation over the coast region of SEC during weak CP El Niño. This may be explained by that the anomalous anticyclone does not cause strong anomalous southwesterlies over the coast region of SEC. The anomalous southwesterlies over the north of SEC is linked to the westerly anomalies to the south of Tibetan Plateau. These analyses further confirm that the strong anomalous anticyclone over the WNP is a key factor, by which El Niño affects precipitation over SEC.

To further verify the importance of anomalous anticyclone over the WNP in precipitation anomalies in SEC, we composite 850-hPa wind anomalies for positive and negative anomalies of precipitation in SEC for weak El Niño events (Fig. 3e,f). There is a strong anomalous anticyclone over the WNP for weak El Niño when precipitation over SEC is above normal (1963, 1968, 1994, 2002, 2004). However, there is no apparent anomalous anticyclone over the WNP when precipitation over SEC is below normal (1965, 1969, 1976, 1977, 1979, 1986, 1987, 2006, 2014). These features are also true when only weak CP El Niño events are considered.

**Figure 4:**
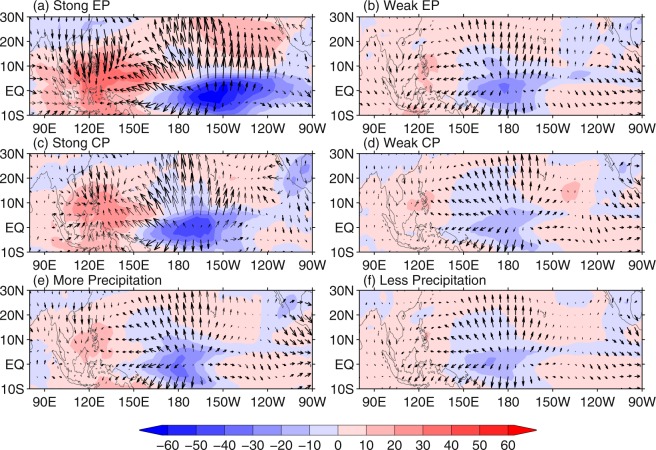
Composite anomalies of DJF OLR (W/m^2^; shadings) and 200-hPa divergent wind (m s^−1^; vectors) for (**a**) strong EP El Niño, (**b**) weak EP El Niño, (**c**) strong CP El Niño, and (**d**) weak CP El Niño. Composite anomalies of DJF OLR (W/m^2^; shadings) and 200-hPa divergent wind (m s^−1^; vectors) for (**e**) more and (**f**) less precipitation in SEC for weak El Niño events. The bold arrows indicate values exceeding 90% confidence level in either the zonal or the meridional component.

Previous studies indicated that the anomalous anticyclone over the WNP is a Rossby wave response to cold SST anomalies and adiabatic cooling of suppressed convection over the WNP, which can be amplified by remote atmospheric forcing^6^. Figure 3a,c show that there are large cold SST anomalies in the WNP for the strong EP and CP El Niño. Meantime, convection over WNP is largely suppressed, which can be partly attributed to the local cold SST anomalies. Besides, there are very warm SST anomalies over the central Pacific, accompanied by local enhanced convection. The upper-tropospheric outflow associated with the enhanced convection over the central tropical Pacific converges over the WNP, and the resultant descending motion suppresses convection over the WNP (Fig. 4a,c). Strong (weak) adiabatic cooling associated with the suppressed convection excite strong (weak) anomalous anticyclone over the WNP^16^. These features suggest that both the central tropical Pacific warm SST and the WNP cold SST anomalies favor suppressed convection over the WNP. Cold SST anomalies in the WNP can be initiated by the surface equatorward northeasterlies excited by warm SST anomalies in the central Pacific, then amplified and maintained by local air-sea interaction in the WNP^6^. This feature is well presented in the composites for strong EP El Niño. However, wind anomalies over the central and western Pacific for strong CP El Niño do not support that the cold SST anomalies in the WNP are caused by the warm SST anomalies over the central tropical Pacific, as there are equatorward northwesterlies over the WNP rather than northeasterlies which are supposed to be at the northwest of a cyclonic circulation over the central tropical Pacific as a response to the warm SST anomalies.

As for weak EP El Niño, there are weak cold SST anomalies in the WNP and warm SST anomalies in the central and eastern tropical Pacific. The convection anomalies over the WNP and the central tropical Pacific are also weak, especially the former (Fig. 4b). In addition, there is no upper-tropospheric convergence over the WNP, similar to the feature of composite for below-normal precipitation (Fig. 4f). Both the SST and convection anomalies do not favor an anomalous anticyclone over the WNP (Fig. 3b). As for weak CP El Niño, there are significant cold SST anomalies in the WNP and warm SST anomalies in the central tropical Pacific, accompanied by an anomalous anticyclone over the WNP (Fig. 3d). Convection anomalies over the WNP and central tropical Pacific are weak, while parts of the upper-tropospheric divergent winds over the central tropical Pacific move northwestward and converge over the WNP (Fig. 4d), similar to the feature of composite for above-normal precipitation (Fig. 4e). Magnitude of both the WNP cold and central tropical Pacific warm SST anomalies for weak CP El Niño is larger than that for weak EP El Niño. Comparison of the anomalous patterns of SST, OLR, 850-hPa winds, and 200-hPa divergent winds between weak CP El Niño and weak EP El Niño indicates that the cold SST anomalies in the WNP may be mostly responsible for the anomalous anticyclone in the WNP for CP El Niño. The magnitude of weak anomalous anticyclone over the WNP for CP El Niño may be attributed to the weak warm SST and convection anomalies in the central tropical Pacific, because model simulations indicated that the warm SST in the central tropical Pacific is responsible for one half of the magnitude of the anomalous anticyclone over the WNP^6^.

**Figure 5:**
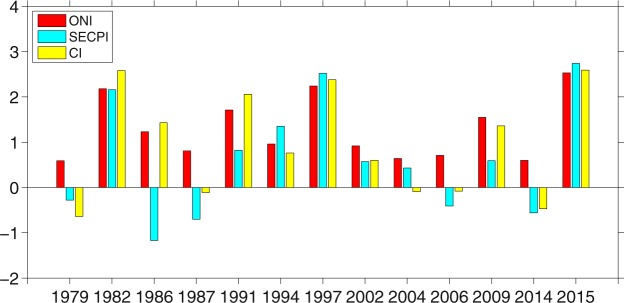
DJF ONI, southeastern China precipitation index (SECPI) and convection index (CI) for El Niño events from 1979 to 2015.

The above analyses suggest that the SST anomalies can affect the magnitude of the anomalous anticyclone over the WNP by modulating the convection over the central tropical Pacific, which induces upper-tropospheric divergent winds convergence over the WNP and thus suppresses convection over there. From this perspective, the convection anomalies over the central tropical Pacific may have a closer relationship with precipitation anomalies over SEC compared to SST anomalies. Figure 5 shows ONI, SECPI, and convection index (CI), where CI represents normalized averaged OLR anomalies over the central tropical Pacific (170°W-150°W, 2.5 S°-2.5°N) multiplied by −1. It can be seen that the convection over the central tropical Pacific is a better indicator for precipitation anomalies over SEC compared to SST anomalies, especially for weak El Niño (Fig. S1). Thus, OLR anomaly over the central tropical Pacific is a considerable factor for the impact of El Niño on precipitation anomalies in SEC.

### Model simulations

The sample size of El Niño events is small, especially for strong El Niño events, which may affect the robustness of the above results. To improve the robustness of the observed results, we analyze the simulations of National Centers for Environmental Prediction (NCEP) Climate Forecast System version 2 (CFSv2)^26^. El Niño events in NCEP CFSv2 simulations are shown in Table S1. Both the coupled and the atmosphere-only simulations show that precipitation increases significantly over SEC during strong El Niño, while precipitation anomalies over SEC show large uncertainty for weak El Niño. The difference of precipitation anomalies over southeastern China between strong and weak El Niño is more apparent for the EP El Niño compared to the CP El Niño (Figs S2 and S3), which is consistent with the observation (Fig. 2).

Figures S4 and S5 show composite 850-hPa wind anomalies for different group of El Niño. It can be seen that significant anomalous anticyclone over the WNP is found for all strong El Niño. A weak anomalous anticyclone over the WNP is also found for weak CP El Niño, especially in the atmosphere-only simulations. All these features are basically similar to those in the observation (Fig. 3).

## Conclusion and Discussion

Southeastern China receives considerable precipitation in boreal winter. Previous studies reported that boreal winter precipitation in SEC is linked to ENSO, with asymmetric impacts of El Niño and La Niña on SEC precipitation. Both the EP and CP El Niño events tend to produce above-normal precipitation over SEC in spite of different magnitude of precipitation anomalies. In this study, we find that strong El Niño events are accompanied by above-normal precipitation in SEC for both EP and CP El Niño, but most weak El Niño events are accompanied by below-normal precipitation. The anomalous anticyclone over the WNP is the key circulation system, by which El Niño causes above-normal precipitation over SEC. Both the cold SST anomalies in the WNP and the warm SST anomalies in the central tropical Pacific are important for the formation of anomalous anticyclone over the WNP. The cold SST anomalies in the WNP only excite a weak anomalous anticyclone locally when the warm SST anomalies in the central tropical Pacific are accompanied by weak convection anomalies. The OLR anomaly over the central tropical Pacific should be considered for the impact of El Niño on precipitation anomaly in SEC.

While the impact of El Niño with different intensity on precipitation anomaly in SEC is different, the composite precipitation anomaly in SEC is below normal for both weak and strong La Niña events (figures not shown). Warm SST anomalies are also found in the eastern tropical Indian Ocean during strong El Niño events (Fig. 3a,c), especially for EP El Niño. These warm SST anomalies may also contribute to the above-normal precipitation anomalies in SEC by inducing a cross-equatorial meridional circulation with anomalous descent over the Philippine Sea^27,28^. As there is a considerable uncertainty in the impact of El Niño on extratropical climate variability over the land^29^ and the precipitation in SEC is also affected by extratropical atmospheric circulation, the response of precipitation anomalies in SEC to El Niño with similar intensity shows some differences from one case to another. As the classification of intensity and type of El Niño is only based on the winter ONI and El Niño Modoki index (EMI)^30^, respectively, there are some differences in classification between current study and other studies, such as the 1987/88 and 2015/16 El Niño events. However, these differences in classification of El Niño events do not obviously affect the results obtained in this study (figures not shown).

## Methods

### Datasets

The data of observed precipitation used in this study cover the period from December 1961 to February 2016. The 1961 winter (DJF) represents the three months mean from December 1961 to February 1962. Monthly precipitation observations of 753 stations in China are obtained from the National Meteorology Information Center, China Meteorological Administration (NMIC, CMA; http://data.cma.cn/en). The quality control has been processed by NMIC, CMA^31^. Monthly wind at 850-hPa with horizontal resolution of 2.5° × 2.5° data are from the National Centers for Environmental Prediction-National Center for Atmospheric Research (NCEP-NCAR) Global Reanalysis 1 (NCEP-1)^32^. Monthly outgoing longwave radiation (OLR) with horizontal resolution of 2.5° × 2.5° for the period 1979–2016 is from NOAA^33^. The SST data with horizontal resolution of 2° × 2° derives from the merged Extended Reconstructed SST version 3b^34^. The ONI which is defined as mean SSTA in the Niño3.4 region (5°S–5°N, 170°–120°W) derives from the Climate Prediction Center (CPC; http://origin.cpc.ncep.noaa.gov/products/analysis_monitoring/ensostuff/detrend.nino34.ascii.txt) and used as an index to depict the ENSO variability in this study. Climatology represents the period from 1981 to 2010.

The simulations of NCEP CFSv2 are applied in this study. Outputs from two types of simulations including atmosphere-ocean coupled and atmosphere-only by the CFSv2 are used^35^. One is the 9-month atmosphere-ocean coupled simulations from 1982 to 2016, initialized with observed atmospheric and oceanic status in November, having 24 ensemble members. The other is atmosphere-only simulation, forced by observed SST and initialized from January 1950 with 11 different atmospheric initial conditions and ended in December 2010. The climatology of the simulations is the average over the periods of 1982–2010 and 1980–2009 for the coupled and the atmosphere-only simulation, respectively.

### ENSO classification

El Niño events are classified into the strong and weak events respectively according to DJF ONI. The strong El Niño events are identified with the DJF ONI reaching up to 1.5 °C, being about 1.3 standard deviation of ONI. Otherwise, the rest is classified as the weak El Niño events. The intensity criterion of 1.5 °C is same as that on website https://www.ggweather.com/enso/oni.htm. There are six strong events in the years 1972/73, 1982/83, 1991/92, 1997/98, 2009/10 and 2015/16, including three super strong El Niño events as 1982/83, 1997/98 and 2015/16^24,36^ and fourteen weak El Niño events. The EMI is used to distinguish CP type El Niño event from EP type El Niño. There are 9 El Niño events and 11 CP El Niño events identified during the study period, respectively (Table 1). A Student’s *t* test is used to determine the statistical significance of the correlation and composition.

**Table 1:** Yeas of El Niño events for different combinations of intensity and type.

Strong EP El Niño	Weak EP El Niño	Strong CP El Niño	Weak CP El Niño
1972/73, 1982/83, 1997/98, 2015/16	1969/70, 1976/77, 1986/87, 1987/88, 2006/07	1991/92, 2009/10	1963/64, 1965/66, 1968/69, 1977/78, 1979/80, 1994/95, 2002/03, 2004/05, 2014/15

## Supplementary information

Supplementary Information

## Data Availability

The data associated with this paper is available on request from author Z. J. K.
