# Supplementary material for: Southeastern China Boreal Winter Precipitation Anomalies are Dependent on Intensity of El Niño
Source: Sci Rep. 2019 Nov 22;9:17410. doi: 10.1038/s41598-019-53496-5 (PMC6874680; doi:10.1038/s41598-019-53496-5)
Supplement: Supplementary file 1 — Supplementary Information [file 41598_2019_53496_MOESM1_ESM.pdf]

Southeastern China Boreal Winter Precipitation Anomalies are Dependent on Intensity of El Niño

Zongjian Ke[1], Xingwen Jiang[2*] & Zunya Wang[1]

[1] Laboratory for Climate Studies, National Climate Center, China Meteorological Administration, Beijing, China

[2]Institute of Plateau Meteorology, China Meteorological Administration, Chengdu, Sichuan, China

Supplementary information

Table S1. Categories of El Niño events in NCEP CFSv2 simulations.

	Strong EP El Niño	Weak EP El Niño	Strong CP El Niño	Weak CP El Niño
Coupled Simulation	1982/83,1997/98, 2015/16	1986/87,1987/88, 2006/07	1991/92, 2009/10	1994/95,2002/03, 2004/05, 2014/15
Atmosphere-only Simulation	1972/73,1982/83,1997/98	1951/52,1953/54, 1969/70,1976/77, 1986/87,1987/88, 2006/07	1957/58,1991/92, 2009/10	1958/59,1963/64, 1965/66,1968/69, 1977/78,1979/80, 1994/95, 2002/03, 2004/05

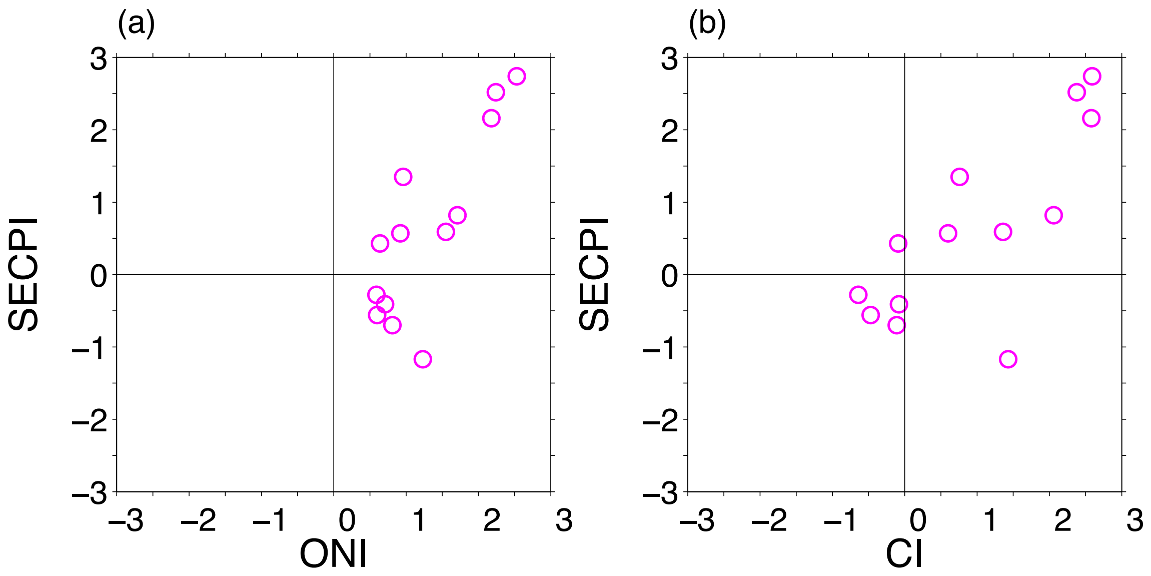

Figure S1. Scatterplot of SEC precipitation with (a) ONI, and (b) convection anomalies index over the central Pacific (CI).

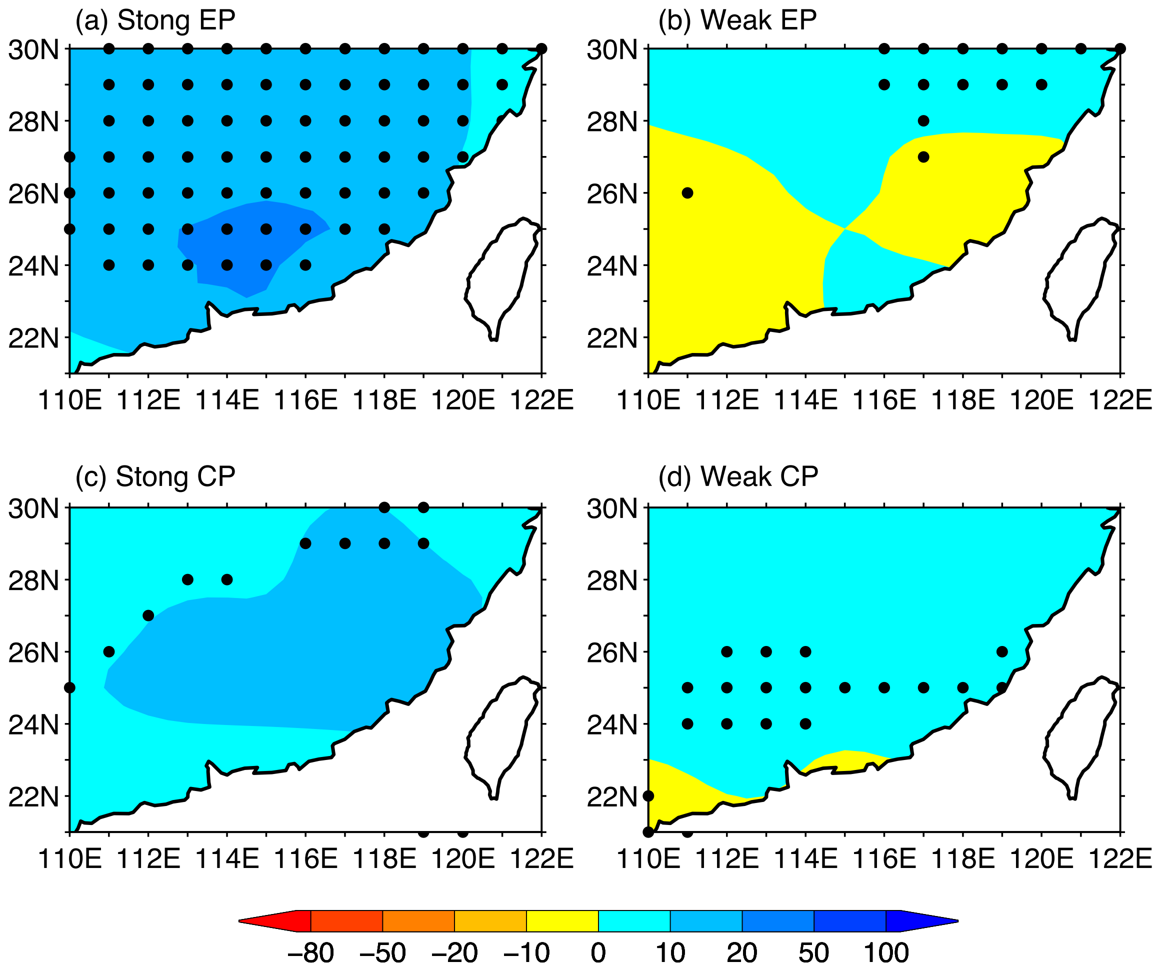

Figure S2. Composite percentage of DJF precipitation anomalies from NCEP CFSv2 coupled simulations. (a) strong EP El Niño, (b) weak EP El Niño, (c) strong CP El Niño, and (d) weak CP El Niño. Stippling indicates the value of shadings exceeding 90% confidence level.

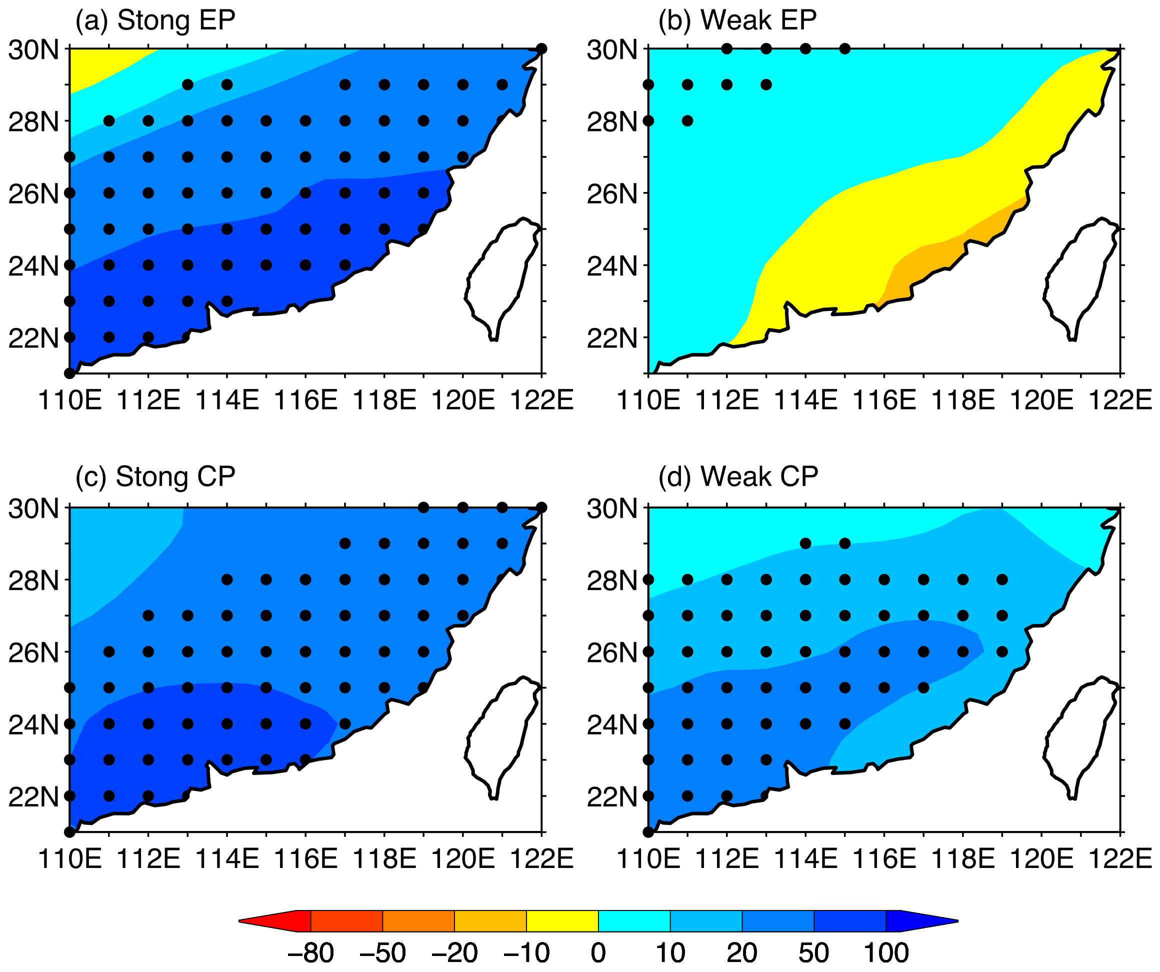

Figure S3. Composite percentage of DJF precipitation anomalies from NCEP CFSv2 atmosphere-only simulations. (a) strong EP El Niño, (b) weak EP El Niño, (c) strong CP El Niño, and (d) weak CP El Niño. Stippling indicates the value of shadings exceeding 90% confidence level.

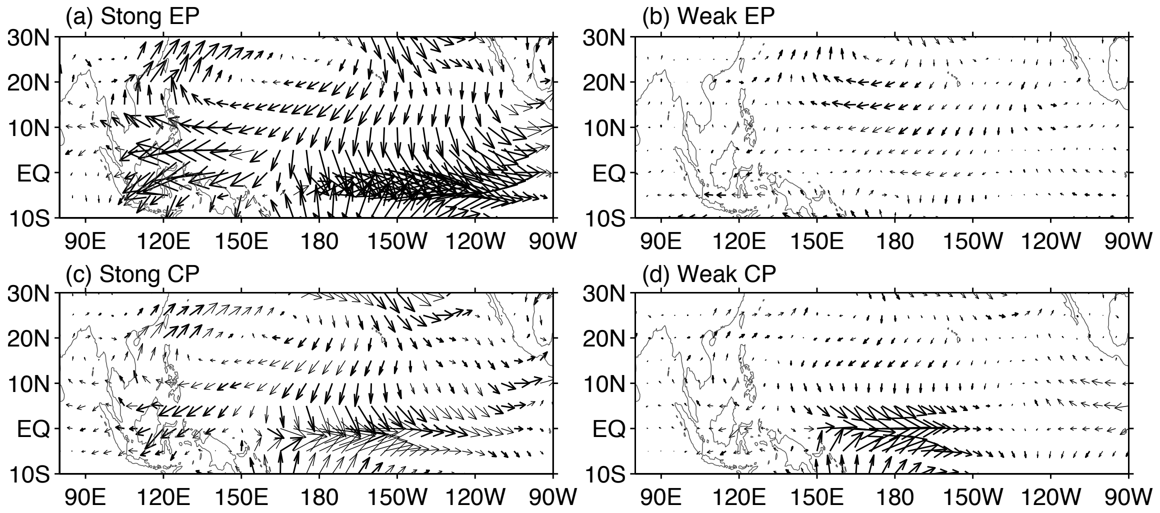

Figure S4. Composite anomalies of DJF 850-hPa wind (m s^{-1}; vectors) from NCEP CFSv2 coupled simulations. (a) strong EP El Niño, (b) weak EP El Niño, (c) strong CP El Niño, and (d) weak CP El Niño. The bold arrows indicate values exceeding 90% confidence level in either the zonal or the meridional component.

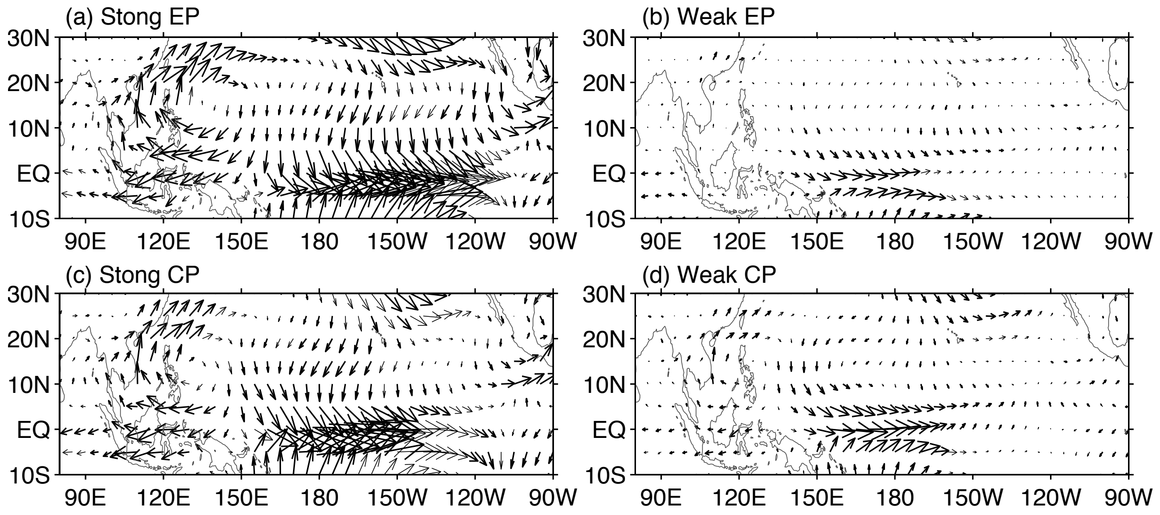

Figure S5. Composite anomalies of DJF 850-hPa wind (m s-1; vectors) from NCEP CFSv2 atmosphere-only simulations. (a) strong EP El Niño, (b) weak EP El Niño, (c) strong CP El Niño, and (d) weak CP El Niño. The bold arrows indicate values exceeding 90% confidence level in either the zonal or the meridional component.
